# Stress and the Role of the Gut–Brain Axis in the Pathogenesis of Schizophrenia: A Literature Review

**DOI:** 10.3390/ijms22189747

**Published:** 2021-09-09

**Authors:** Behnam Vafadari

**Affiliations:** Clinic for Anesthesiology, University Medical Center Göttingen, Georg-August-University, 37073 Göttingen, Germany; behnam.vafadari@med.uni-goettingen.de

**Keywords:** schizophrenia, neuropsychiatric, stress, gut–brain axis, psychosocial

## Abstract

Schizophrenia is a severe neuropsychiatric disorder, and its etiology remains largely unknown. Environmental factors have been reported to play roles in the pathogenesis of schizophrenia, and one of the major environmental factors identified for this disorder is psychosocial stress. Several studies have suggested that stressful life events, as well as the chronic social stress associated with city life, may lead to the development of schizophrenia. The other factor is the gut–brain axis. The composition of the gut microbiome and alterations thereof may affect the brain and may lead to schizophrenia. The main interest of this review article is in overviewing the major recent findings on the effects of stress and the gut–brain axis, as well as their possible bidirectional effects, in the pathogenesis of schizophrenia.

## 1. Introduction

Around 792 million people in the world live with mental disorders, which is more than one in ten people (10.7%) globally [1]. Neuropsychiatry is a discipline that deals with mental disorders that are attributable to diseases of the nervous system; these are related to cognitive and behavioral diseases caused by direct cerebral dysfunctions or indirect extra-cerebral diseases [2]. In spite of major advances in neurobiology and neuroscience research, the prevalence of neuropsychiatric diseases has not decreased. Our knowledge in the basic and clinical pathophysiology of neuropsychiatric diseases, such as schizophrenia, is still limited. “Schizophrenia is a severe neuropsychiatric disease that is characterized by impairments in perception, cognition, and avolition, leading to positive, cognitive, and negative symptoms, respectively” [3]. The etiology of schizophrenia remains unknown [4]. As a result, the objective of this review is to assess the newly emerging paradigm of the brain–gut–microbiota axis, in which imbalances are risk factors for the pathogenesis of schizophrenia, in addition to the effects of other environmental factors (e.g., stress) on the pathogenesis of schizophrenia. Several studies focused on the risk factors for schizophrenia separately, and we mention the important findings in this review article. For example, there is increasing evidence that disorders such as schizophrenia are associated with an imbalance in the gut microbiome [5,6]. Another important factor is stress. Stress can induce changes in the gut microbiome [7] and it is well known that stress is one of the risk factors for the pathogenesis of schizophrenia [3,8,9,10,11]. Finally, several lines of evidence support the notion that gut microbiota can affect stress-related behaviors [12].

**Figure 1:**
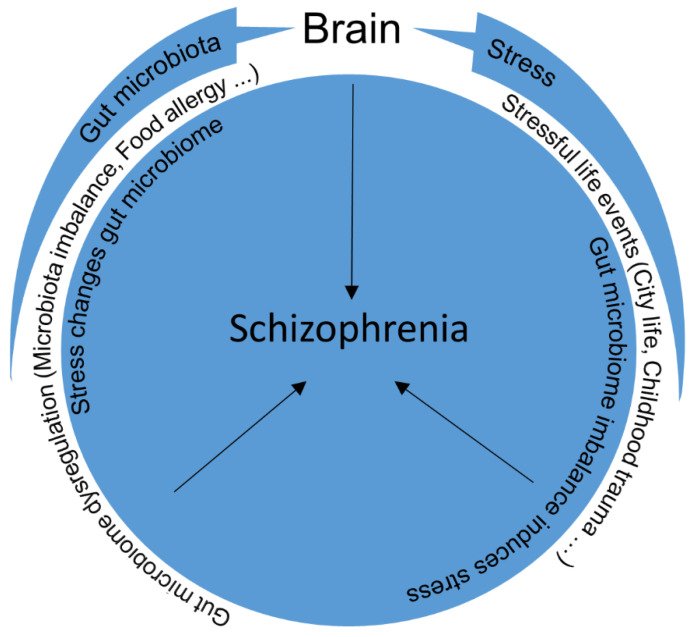
Stress and dysregulation of the gut microbiome can rarely be effective alone. Stressful life events in combination with the effects of the gut–brain axis may induce bidirectional effects and lead to schizophrenia.

The effects of environmental factors (stress) together with those of the dysregulation of the gut–brain axis on the pathogenesis of schizophrenia will be discussed in this review (Figure 1). This can be interesting due to the fact that there are few studies that show the bidirectional effects of these factors. Some of the important studies, summarized in Table 1.

## 2. Schizophrenia

Schizophrenia is a severe neuropsychiatric disorder that affects 20 million people worldwide [13]. Schizophrenia causes a significant burden on wellbeing and global health. The interactions of different factors that play roles in the disease’s etiology are not fully understood. There are possible reasons for the onset of schizophrenia, such as the possible increased activation of components of the immune system [14], including alterations in intestinal permeability and the gut microbiome [15], and stressful life events [8,9,10,11]. There are three major groups of symptoms recognized in schizophrenia, which are classified as positive, negative, and cognitive [16]. The etiology of schizophrenia remains largely unknown, although the roles of the genes in combination with environmental factors have been reported [17,18]. The onset of schizophrenia is typically in early adulthood, and it is associated with life time disability [19,20,21]. Dysfunctions in dopaminergic neurotransmitters may contribute to psychotic symptoms, although there is evidence that shows the involvement of other areas and circuits of the brain [22].

The terminology for positive and negative symptoms has been evolved for around 150 years [23].

Clinically, schizophrenia is characterized by its diverse psychopathology; the core features are positive symptoms (thought disorders, delusions and hallucinations, and psychotic symptoms in which the person loses contact with reality), negative symptoms (restricted affect, poverty of speech, impaired motivation, and social withdrawal), and cognitive impairments (impairments in cognitive processes, such as memory dysfunction, poor performance in a wide range of cognitive functions, working memory deficits, executive dysfunction, and attentional impairments) [3,24,25,26,27]. Importantly, a patient with cognitive symptoms may show a cognitive disability; however, a patient with negative symptoms may just show either anhedonia or unsociability without other signs. Furthermore, each of those symptoms can be individually scaled [27]. The heterogeneity of the symptoms of the disease makes it very difficult to diagnose and cure patients. As a result, the etiology of schizophrenia is not yet fully understood. The disorder’s etiology is multifactorial; as a result, investigation of the different risk factors (e.g., gut microbiome dysregulation and stress) alone or in combination can be very helpful.

## 3. Stress

Psychological stress can lead to several neuropsychiatric disorders, such as depression and schizophrenia [28]. Nowadays, it is suggested that environmental factors may play a major role in the pathogenesis of schizophrenia. Epidemiological results have demonstrated specific circumstances in which the risk for schizophrenia is increased. These circumstances include migration, adversity in early life, growing up in an urban environment and urban residence, and position in a minority group [11], can be interpreted as stressful situations.

Interestingly, several studies have suggested that stressful life events, especially city life, may lead to schizophrenia [8,9,10,11]. Furthermore, the risk for schizophrenia is increased with urban birth and/or upbringing [9]. Childhood trauma is another important environmental factor that can increase the risk for the development of schizophrenia in adulthood [29]. It is known that schizophrenic patients are exposed more to early life trauma than the controls; for example, children who were raised in dysfunctional adoptive families and institutions [30,31]. Stress animal models can be proper models to study the neurodevelopmental factors in the pathogenesis of schizophrenia, as well [32]. There are animal studies that have shown the role of stress in the pathogenesis of schizophrenia. For instance, ref. [33] showed the role of maternal deprivation in animal models of schizophrenia. In another study, ref. [34] used a maternal malnutrition model and a Prepulse inhibition test (PPI) in rat models. The PPI was reduced in females with age, which is a clinical symptom of schizophrenia [35]. Ref. [3] used adolescent matrix metalloproteinase-9 (*Mmp-9*) heterozygous mice (after weaning) that were chronically subjected to psychosocial resident–intruder stress and then examined by using behavioral tests. In those mice, negative symptoms were manifested after exposure to stress. Interestingly, after clozapine (atypical antipsychotic) administration, the negative symptoms were ameliorated.

Stress can rarely be effective alone, and it usually acts in parallel with one or more other factors. One example is immune dysregulation caused by stress. Stress can trigger the hypothalamic–pituitary–adrenal axis (HPA), which is an important part of the neuroendocrine system and has a role in regulating the immune system and mood [36]. Evidence of HPA dysfunction also exists in schizophrenia [30]. It was also revealed in animal models that maternal separation is an early-life stressor that can lead to long-term increases in HPA activity [37]. Stressful situations can lead to hypothalamic activation, which causes the secretion of corticoids by the adrenal cortex. Several reports showed hypercortisolemia in schizophrenic patients [38]. Maternal stress increases locomotor behavior (amphetamine induced) in the adult offspring (model of psychosis) [30] and in primates, prenatal stress can cause attention impairment, and decreased locomotion as well as hyperreactivity in the adult offspring [39]. The rhesus monkeys, which were under prenatal stress, showed more disturbance behavior as well as dysregulation of the HPA axis [40].

These findings show the fact that chronic stress can contribute to vulnerability to schizophrenia [41].

## 4. Gut–Brain Axis

It is well known that the gut microbiome is the most important regulator of the gut–brain axis. This effect is so prominent that some studies have called human gut microflora the “second brain” [42]. The gut is a niche for microbes—especially bacteria, but also other microorganisms [12]. The gut microbiome mainly consists of two major phylotypes—Bacteroidetes and Firmicutes, and the rest, mostly including Proteobacteria, Actinobacteria, Fusobacteria, and Verrucomicrobia phyla [43].

The bilateral effects of the gastrointestinal tract on brain function have been recognized since the nineteenth century [44]. Our knowledge about the gut microbiome and its effect on brain function has been expanded over the last decade. The microbiome has been introduced as an important cause for neurological disorders according to clinical and preclinical reports [45]. The importance of this bilateral homeostatic communicational route that uses neural, hormonal, and immunological routes—dysfunctions of which can lead to pathophysiological consequences—is still gaining recognition [46]. Dysfunctions in brain–gut interactions can be associated with eating disorders and gut inflammation. It is also clear that the modulation of gut–brain interaction can be associated with stressful behaviors. Stress-related psychiatric symptoms, such as stressful behaviors, and gastrointestinal disorders, such as irritable bowel syndrome, show the significant importance of the pathophysiology of the brain–gut axis [44,46,47].

Communication between gut microbes and the brain is possible through the gut–brain connection, and this may cause microbiota to be modulators of the brain and behavior [47,48]. There is communication between gut microbes and centrally or peripherally mediated behavior. This communication can be facilitated by the vagus nerve. A central *Lactobacillus rhamnosus* deletion was shown through a full truncal vagotomy. Similarly, patients with peptic ulcers showed a lower risk for neurological disorders, such as Parkinsonism, in old age after a full truncal vagotomy [49,50]. Apart from nerves, gut microbes can regulate neurotransmitters by changing the precursor levels (e.g., serotonin). Serotonin (5-HT) is a key neurotransmitter in the gut–brain axis communication system, and it functions between the central nervous system and the gastrointestinal tract [51]. The precursor of 5-HT is tryptophan, however, it has limited storage in the brain. As a result, intestinal refilling is crucial. This can be performed with *Bifidobacterium infantis*. There are also bacteria that can produce and release different neurotransmitters. For instance, acetylcholine, dopamine, 5-HT, and norepinephrine can be produced and released by the Lactobacillus, Bacillus, and Enterococcus species, the Candida, Streptococcus, Escheridia, and Saccharomyces species, and the Escheridia and Bacillus species, respectively [52,53,54]. These interactions are also very important for mental health and sociability [5,55]. It was revealed through oral microbiome analyses that people with schizophrenia had different levels of Lactobacillus phage phiadh. The presence of immunological conditions was correlated with different levels of Lactobacillus phage phiadh [56].

Several animal studies have shown the role of the gut–brain axis in psychiatric illnesses [57]. In one study, it was shown that adolescence and early adulthood can be critical periods in which the dysregulation of the communication along the microbiota–gut–brain axis can significantly impact brain development and behavior. This can lead to alterations in cognitive and anxious phenotypes in adulthood. Additionally, chronic antibiotic treatment is a useful model for revealing the importance of gut microbiota during early stages of life in brain development and behavior [58]. The gut–brain axis and microbiome have potential roles implicated in schizophrenia pathogenesis [59,60]. Additionally, gut microbiome changes may contribute to schizophrenia pathophysiology and behavioral symptoms development [61]. Increasing evidence has shown that disorders such as schizophrenia are associated with gut microbiome imbalances [5,6]. For example, ref. [62] showed that the schizophrenic patients gut microbiome can modulate the glutamate-glutamine-GABA cycle and schizophrenia-like behaviors in mice. Similarly, ref. [63] revealed that drug free schizophrenic patients microbiota transplantation can lead to the mice schizophrenia-like behaviors by tryptophan-kynurenine metabolism dysregulation. Additionally, transplantation of Streptococcus vestibularis (schizophrenia-enriched bacterium) can cause social behavior deficits and change the peripheral tissues neurotransmitters levels in mice [64]. In another study, ref. [65] reported that patients with schizophrenia had decreased oral microbial biodiversity. In this study, they used MaAsLin to detect the effect of schizophrenia on the composition of microbiome species while considering the effects of other variables (e.g., medication) in the studied population. They found that the overall microbial composition in schizophrenic patients was significantly different from that in the non-schizophrenic subjects [65]. Finally, ref. [66] showed that human gut microbiota transplantation might improve the treatment of the schizophrenia.

The composition of gut microbiota may affect the gastrointestinal barrier, immune regulation, and metabolism associated with schizophrenia [60]. Schizophrenia is a brain disorder; therefore, to assess the gastrointestinal system’s role in the etiology of schizophrenia, mechanisms that can affect the brain should be included. Toxic products can exit the gastrointestinal tract, which may cause an immune response by entering the brain; thus, these products need to penetrate the barriers in the gastrointestinal tract, as well as the barriers of the blood and central nervous system [67]. We know that the microbiome can modulate the immune response [68]. A microbial imbalance can cause an inflammatory response that is followed by immune reactions and vice versa. In one study, ref. [69] used the maternal immune activation by poly I:C injection. Afterwards, they studied the behavioral changes relevant to the gut–brain axis in the offspring of an outbred NIH Swiss and an inbred C57BL6/J mouse strain. They revealed that this can cause social deficits in both strains.

Alterations in the gut microbiome have been linked to the pathogenesis of allergic, neurodevelopmental, psychiatric, and neurodegenerative diseases [70].

Environmental factors, such as microbial exposure, have been introduced as possible factors that cause allergic diseases [71]. The reason is still unknown, however, a possible cause can be the microbiota that colonize the gut of infants. Recent studies have shown that there are imbalances in the intestinal microbial flora of children and infants with food allergies [72]. A typical food that can lead to different psychological states is bread. Bread can make the gut permeable and, therefore, cause the migration of food allergens to areas in which they should not be. This causes the immune system to attack these allergens, as well as brain-related substances [73]. Similarly, the availability of wheat has been correlated with hospitalization rates for schizophrenia [74]. It is known that gluten can be broken into bioactive opioid receptor peptides, which can enter the gastrointestinal tract, as well as brain barriers [75]. Another condition called gluten sensitivity has also been shown to have an association with schizophrenia [76]. Increases in other food antigens, such as milk caseins, have also been reported in schizophrenic patients [67]. Autopsies of schizophrenic patients revealed that 50% of them had gastritis, 88% had enteritis, and 92% had colitis [77,78]. Additionally, there is a well-known association of schizophrenia with celiac disease [67].

## 5. Stress and Gut Microbiome

The stress and gut microbiome interaction is an important topic. Stress and diet, together or independently, can influence the gut microbiome. On the other hand, the gut microbiome can modulate stress and mood. Finally, the stress and microbiome interactions can affect health [79]. It is known that the gut microbiome composition can be changed by stressors. The microbiome role in stress response regulation was also shown [80]. There are several effects of stress on gut function: alterations in gastrointestinal motility, increases in visceral perception, alterations in gastrointestinal secretion, changes in intestinal permeability, and dysregulation of intestinal microbiota. Gastrointestinal physiology can be affected by pro-inflammatory cytokines and different kinds of neurotransmitters. These can be released by stress signals translated by mast cells [7]. For example, in rhesus monkeys, maternal separation led to a decrease in fecal lactobacilli [81]. In adult rats, stress in early life may induce long-term effects on the composition of gut microbiota by causing the elevation of the circulating levels of interleukin-6 and the chemokine CCL2. These have been correlated with changes that are induced by stressors in the levels of three bacterial genera: Pseudobutyrivibrio, Coprococcus, and Dorea. This may show that repeated stress affects the populations of gut bacteria, and these changes are correlated with alterations in the levels of pro-inflammatory cytokines [44,82]. Microbiota can also influence the priming and recovery of the innate immune system with respect to an acute stressor [83]. Ref. [84] showed an association of the human infant gut microbiome with fearful behavior and the microbiome’s relationship with fear-related brain structures. On the other hand, the effects of the gut microbiome on the brain and stress have been reported in animal studies. According to these studies, the gut microbiome may affect stress-related behaviors, such as those related to depression and anxiety. For example, germ-free mice experiments showed a link between the microbiome and anxiety-like behavior [85,86,87,88]. Similarly, ref. [89] investigated if penicillin administration in early postnatal life induces long-term effects on the offspring of mice. They showed that there were changes in gut microbiota, increases in cytokine expression in the frontal cortex, anxiety-like behaviors, and impaired social behaviors [89]. The fearful behaviors and related neurocircuitry was changed in animal models, which their gut microbes manipulated [84]. On the other hand, early life stress can also lead to the gut–brain axis alterations that may contribute to stress-related and psychiatric disorders development in adulthood [37]. It is also known, as well, that gut microbiome chronic change (i.e., being under long time stress) may lead to neuroplastic alterations [32].

## 6. Stress, the Gut, and Schizophrenia

Research on animal models has been used to discuss the possible effects of the gut microbiome on immune changes in the central nervous system and behavior, as well as their possible contributions to the pathophysiology of psychiatric disorders. Early life events may change the gut microbiome and can cause psychosis development later [61]. Early life stress can cause long term gut microbiome alterations, and may lead to neuronal malfunction and behavioral changes which have a role in psychiatric disorders. One study was about behavioral changes after weaning under social isolation in rats. The composition of the microbiota of these rats was altered with an elevation of Actinobacteria and decrease in Clostridia. The positive correlations were reported for open-field exploration, conditioned freezing, microbiota, and hippocampal IL-6 and IL-10 [90]. This may show alterations in the gut microbiota due to early-life stress. The production of different cytokines in the hippocampus can contribute to the abnormal neuronal functions and behavior development, which may lead to the onset of psychiatric illnesses [90].

The effects of dysfunction in the gut microbiome on the brain can be caused by stressful situations; otherwise, inflammation can be a strong activation mechanism that affects the brain and makes it more susceptible to psychiatric conditions, such as depression [91] and schizophrenia [60]. We know that stress and schizophrenia can be influenced by the gut microbiome. The HPA axis and the brain in kids can be affected by several factors such as stress and infection. During the development, several stressors such as physical and psychic trauma, inflammations, and metabolic dysregulations may lead to microbiome changes. These interactions of microbial and neurological pathways can affect each other during the development. Early childhood trauma can affect the gut microbiome and this may influence the risk of schizophrenia [92]. There are studies which show the possible connection between stress, the gut, and schizophrenia. It was shown in animal models that several types of stressors can change the gut microbiome composition. On the other hand, the important role of the microbiome in regulating the stress response was also shown in animal models [80]. For example, ref. [93] showed that gut microbiome nurturing with prebiotics such as galacto-oligosaccharides and fructo-oligosaccharides can reduce chronic stress as well as depression-like behavior in mice. Similarly, several reports showed gut microbiome alterations in major depressive disorder animal models and patients [94]. Interestingly, depressive symptoms can be common clinical features of schizophrenic patients [94,95].

Other studies, which can show the connections between gut microbiome, stress, and schizophrenia, are about effects of the gut microbiome on brain structures and neural circuits which can be implicated in schizophrenia pathogenesis. Ref. [96] showed associations between some brain structures and the gut microbiome in schizophrenia patients. Furthermore, microbial deficiencies can change stress-related neurotransmitters in the related brain regions [12]. One of these regions is the amygdala, which is the key brain region to process anxiety [97] and can modulate fear responses [98]. Altered processes in the amygdala are associated with both stress [99] and neuropsychiatric disorders, such as schizophrenia [100,101]. Ref. [97] showed that the microbiome can regulate amygdala-dependent fear memory in germ free mice. In another study, ref. [102] investigated the gut microbiome and brain functional connectivity, focusing on the amygdala. They also demonstrated the associations between the gut microbiome and neural circuits integrity, the circuits which are important for cognition and fear processing. These two can be associated with future psychopathology vulnerability. Another brain region which dysfunction is deeply implicated in neuropsychiatric disorders such as schizophrenia is the prefrontal cortex [19,103,104,105,106]. The prefrontal cortex has an important role in anxiety and fear processes. It has been implicated in the HPA axis regulation as well [107,108]. Ref. [105] showed that the microbiome has an important role in the prefrontal cortex myelination in germ free mice and can be used as a therapeutic target for psychiatric disorders. In another study, it was shown that the gut microbiome can modify the key metabolites synthesis which are affecting the prefrontal cortex gene expression [109]. Furthermore, gut microbiome absence can influence the mice prefrontal cortex lipid metabolism [110].

As it was described earlier, both the amygdala and prefrontal cortex are regions, which are implicated in schizophrenia pathogenesis [111,112,113,114]. These are also two regions involved in controlling anxiety and fear [115,116] and the abnormalities (prefrontal cortex hypermyelination and amygdala changes) of these regions were seen in germ free mice [105,117]. Finally, it is known that the miRNA expression regulation in the prefrontal cortex and amygdala can be affected by microbiome activity [116]. Anyhow, the precise role of the gut microbiome together with environmental stressors has not yet been investigated in schizophrenia [118]. The role of the microbiome, which is affected by the environmental factors, such as stress, can be a very interesting topic for researchers. The combination of the microbiome with life stressors is a new topic that can explain features of schizophrenia [92].

## 7. Conclusions

In this review, the author aimed to bring together some recent studies that showed the effects of stress as well as dysregulation of the gut–brain axis on the pathogenesis of schizophrenia. There are not many studies that have shown the precise mechanisms underlying schizophrenic symptoms in human patients or in animal models of schizophrenia. Anyhow, there are several reports showing the symptoms of schizophrenia in patients or animal models after being exposed to environmental factors, such as stress, or models with dysregulations in the gut–brain axis. Each of these factors can play a role in schizophrenia pathogenesis and they may have bidirectional effects. These factors can also have bidirectional effects on the brain. Few studies have shown the bidirectional effects of stress together with the gut microbiome and the possible roles of both effects in the pathogenesis of schizophrenia. Nevertheless, it is important to recognize different symptoms of schizophrenia and the possible reasons that may lead to each symptom. The roles of stress and the gut microbiome in the pathogenesis of schizophrenia can be an interesting topic to investigate in the future, which may shed light on some of the unanswered questions regarding the etiology of schizophrenia.

## Figures and Tables

**Table 1:** Role of stress and the gut–brain axis in the pathogenesis of schizophrenia. Important studies summary.

Reference	Animal Model/Human	Stress/Gut Microbiome	Outcome
[39]	animal model	stress	attention impairment, decreased locomotion and hyperreactivity in the adult offspring
[33]	animal model	stress	role of maternal deprivation
[38]	human	stress	hypercortisolemia in schizophrenic patients
[107,108]	human	both	prefrontal cortex role in anxiety and fear processes
[30,31]	human	stress	children raised in dysfunctional adoptive families and institutions
[30]	animal model	stress	maternal stress increases locomotor behavior in the adult offspring
[34]	animal model	stress	maternal malnutrition model and reduced PPI
[29]	human	stress	childhood trauma
[8,9,10,11]	human	stress	stressful life events, especially city life
[76]	human	gut microbiome	gluten sensitivity
[65]	human	gut microbiome	different microbial composition in schizophrenic patients
[105]	human	both	important role of the microbiome in prefrontal cortex myelination
[105,117]	animal model	both	abnormalities in prefrontal cortex and amygdala changes were seen in germ free mice
[5]	human	gut microbiome	disorders such as schizophrenia are associated with an imbalance in the gut microbiome
[90]	animal model	both	alterations in the gut microbiota due to the early-life stress
[97]	animal model	both	microbiome can regulate amygdala-dependent fear memory in germ free mice
[62]	animal model	gut microbiome	the schizophrenic patients gut microbiome modulates the glutamate-glutamine-GABA cycle and schizophrenia like behaviors in mice
[3]	animal model	stress	psychosocial stress induces schizophrenia-like behavior in genetically modified Mice
[80]	animal model	both	stressors can change the gut microbiome composition
[92]	human	both	the early childhood trauma can affect the gut microbiome and this may influence the risk of schizophrenia
[66]	human	gut microbiome	human gut microbiota transplantation, might improve the treatment of the schizophrenia
[64]	animal model	gut microbiome	transplantation of Streptococcus vestibularis (schizophrenia-enriched bacterium) can cause the social behaviors deficits in mice
